# Virtual prey with Lévy motion are preferentially attacked by predatory fish

**DOI:** 10.1093/beheco/arad039

**Published:** 2023-05-18

**Authors:** Christos C Ioannou, Luis Arrochela Braga Carvalho, Chessy Budleigh, Graeme D Ruxton

**Affiliations:** School of Biological Sciences, Life Sciences Building, 24 Tyndall Avenue, University of Bristol, Bristol BS8 1TQ, UK; School of Biological Sciences, Life Sciences Building, 24 Tyndall Avenue, University of Bristol, Bristol BS8 1TQ, UK; School of Biological Sciences, Life Sciences Building, 24 Tyndall Avenue, University of Bristol, Bristol BS8 1TQ, UK; School of Biology, University of St Andrews, Sir Harold Mitchell Building, Greenside Place, St Andrews KY16 9TH, UK

**Keywords:** Brownian motion, Lévy flight, Lévy walk, *Gasterosteus aculeatus*, search behavior, three-spined sticklebacks

## Abstract

Of widespread interest in animal behavior and ecology is how animals search their environment for resources, and whether these search strategies are optimal. However, movement also affects predation risk through effects on encounter rates, the conspicuousness of prey, and the success of attacks. Here, we use predatory fish attacking a simulation of virtual prey to test whether predation risk is associated with movement behavior. Despite often being demonstrated to be a more efficient strategy for finding resources such as food, we find that prey displaying Lévy motion are twice as likely to be targeted by predators than prey utilizing Brownian motion. This can be explained by the predators, at the moment of the attack, preferentially targeting prey that were moving with straighter trajectories rather than prey that were turning more. Our results emphasize that costs of predation risk need to be considered alongside the foraging benefits when comparing different movement strategies.

## Teaser

How animals search their surroundings for food and other resources is a key question in biology. Here, we show that when virtual prey use a Lévy search, which has been repeatedly been shown to be highly efficient in encountering resources, the prey are also preferentially attacked by real predators, three-spined sticklebacks. This emphazises the need to consider the cost of different search strategies imposed by predators, as well as the benefits in locating resources.

## INTRODUCTION

Movement to find resources is a general hallmark of the animal kingdom, displayed by most species in at least some life history stages. Describing the trajectories of these movements has long been of interest to biologists (Hansson et al. 2014). A very common theoretical description of such trajectories is a random walk, where the trajectory is broken down into a series of steps, and the characteristics of these steps (specially direction and length) are drawn stochastically from defined probability distributions. Two commonly studied random walks are differentiated by the distribution of step lengths: in Brownian motion, this is a negative exponential, while in Lévy motion, this is a power-law function that is more heavy-tailed than the negative exponential. In both models, most steps are short, but long steps are more common in Lévy than in Brownian motion (Codling et al. 2008; Berg 2018).

Over the last 25 years, Lévy motion has been subject to intense study. Theoretical work suggests that Lévy motion is a highly efficient way to search the environment for hidden resources (Viswanathan et al. 1999; Bartumeus et al. 2002; Guinard and Korman 2021), and trajectories from a broad range of species have been suggested to fit Lévy motion (Sims et al. 2008; Humphries et al. 2012; Raichlen et al. 2014, although see Edwards et al. 2007). However, except for apex predators, how animals move in space also determines encounter rates with, and attractiveness to, their own predators (Huey and Pianka 1981; Yoder et al. 2004; Ioannou and Krause 2009).

Considering encounter rates, it is inevitable that the path taken by a forager through its environment will affect encounter rates with its predators as well as its food. If Lévy motion increases the rate of encounter with a food resource distributed through the environment, then it will also increase the rate of encounter with sit-and-wait predators if they are distributed in the same way. This has been demonstrated in a simulation model by Abe and Shimada (2015). This study further demonstrated that the characteristics of a forager’s trajectory (specifically Lévy or Brownian motion) also affected rates of encounter with mobile predators, and that the relative encounter rate of the two types of trajectory was sensitive to the details of the movement strategy of the predators.

Here, our interest is not in encounter rate with predators, but in the possibility that an animal’s movement might influence post-encounter decision-making by predators. Specifically, if the movement pattern of the prey influences the easy of capture, then we might expect that this would translate into predators showing a preference for attacking some individuals over others on the basis of their movement patterns. Such post-encounter preference for prey has not to our knowledge previously been considered within the paradigm of Lévy motion. It is generally considered that unpredictable movement by prey may hinder capture by predators (Humphries and Driver 1970; Jones et al. 2011; Richardson et al. 2018; although see Szopa-Comley and Ioannou 2022); the lack of turns during longer step lengths, which are more common in Lévy trajectories, may increase the short-term predictability of movement and so increase predation risk. Here we use a system of fish predators (three-spined sticklebacks, *Gasterosteus aculeatus*) targeting computer-generated prey whose motion can be entirely controlled (Duffield and Ioannou 2017; Dobbinson et al. 2020; Lambert et al. 2021) to test the hypothesis that prey with Lévy motion are targeted preferentially relative to prey with Brownian motion, potentially revealing a cost of Lévy motion that counteracts the benefits for finding resources (Viswanathan et al. 1999; Bartumeus et al. 2002; Humphries et al. 2012; Guinard and Korman 2021). Brownian motion was used as the control treatment in comparison to Lévy motion as the contrast between these two movement patterns has been studied extensively in previous studies of animal movement (Bartumeus et al. 2002; Humphries et al. 2010; Sims et al. 2012; de Jager et al. 2013; Abe and Shimada 2015).

## METHODS

### Study subjects and housing

Three-spined sticklebacks were obtained from Carp Co. (www.carpco.co.uk) in September 2021. They were kept in 40 × 70 × 34 cm (width × length × height) glass tanks with a flow-through recirculation system, plastic tubes as shelter and plants for environmental enrichment. They were kept at 14 °C under an 11:13 light: dark cycle and each tank housed a maximum of 100 fish. All subjects in the holding tanks were fed small granular pellets of fish feed every morning except for days of testing when they were fed after the trials were finished.

### Experimental setup

**Figure 1:**
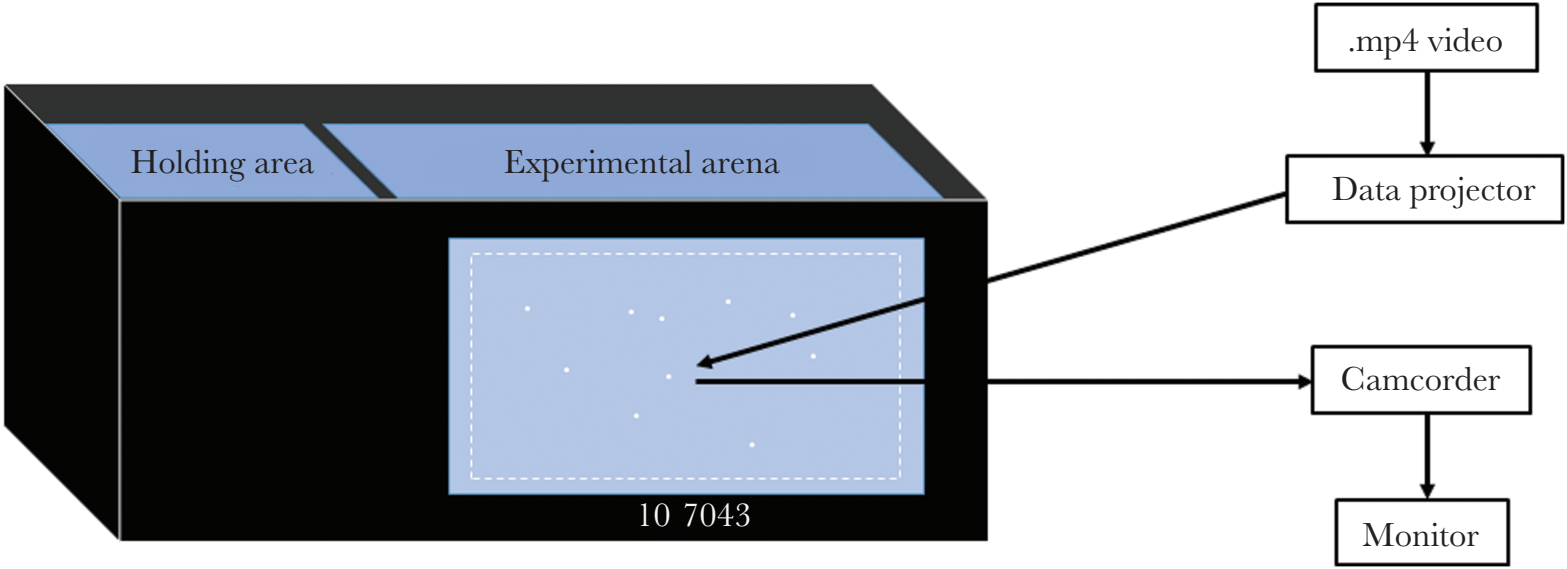
Schematic of the experimental set up, not to scale. The white dashed box, which was not projected during the trials, indicates the area within which the prey could move.

The experiment used a similar setup to other virtual prey experiments (Duffield and Ioannou 2017; Lambert et al. 2021) but had a larger arena in which the virtual prey could move to increase the spacing between the prey, minimizing the prey being perceived as being within the same group. The 36 × 121 × 47 cm (width × length × height) experimental tank (Figure 1) was filled with aged tap water which was continually filtered with an external Eheim Classic 350 filter and chilled with an aquarium chiller (D-D DC300) to maintain the temperature between 13 and 14 °C. The test tank was divided into a 36 × 28 × 47 cm holding area with artificial plants where the fish were habituated the evening before testing, and a 36 × 93 × 47 cm experimental arena where the trials were carried out. A sheet of white translucent plastic film (Rosco gel no. 252) was taped onto the inside of the longest side of the tank facing the projector and camcorder. Black plastic on the outside of the tank framed the translucent screen to create an 84 × 34 cm (width × height) area for the prey to be projected on to (Figure 1). The video number and time step of the simulation were included under the area in which the prey could move to be visible in the view of the camcorder but out of view of the fish. The other walls of the tank were covered internally with black opaque plastic. A strip light was installed above and behind the back wall of the tank to provide illumination so that the fish was visible through the translucent screen, facilitating the detection of attacks by the fish. A data projector (BenQ MW523) was positioned 132.5 cm in front and above the front tank wall and a camcorder was positioned 104.5 cm directly in front of the front wall and below the projector, filming at 1920 × 1080 and 50 frames per second. Due to technical difficulties, Panasonic VX870 and then Panasonic HC-X920 camcorders were used to record the trials. The camcorder was connected to a monitor to remotely observe the trials. Black sheets enclosed the space between the tank and the projector and camcorder in order to provide a dark background against which the fish could view the prey, and to minimize external disturbances.

**Figure 2:**
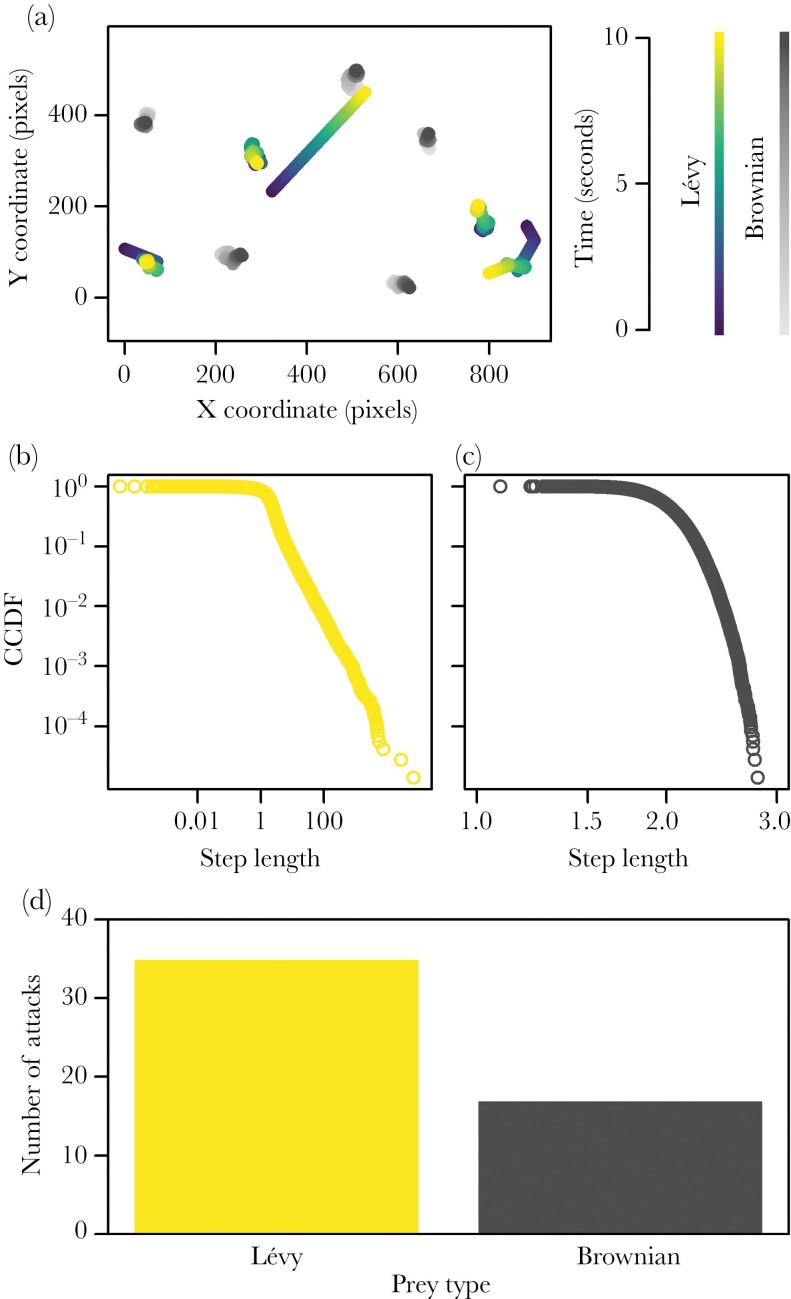
The prey simulation and the number of attacks on each prey type. In (a), the trajectories of the prey over 10 seconds are shown. (b) and (c) show the complementary cumulative distribution function (CCDF) for example Lévy and Brownian trajectories as generated by the Trajr package before these were rediscretised to give a constant speed of movement for each prey (Supplementary Figure S1). (d) shows the number of trials where the first attack targeted each prey type.

The trajectory of each prey was simulated using the TrajGenerate function in the Trajr package (version 1.4.0, McLean and Skowron Volponi 2018) in R version 4.1.2 (Figure 2a) to create trajectories for prey with a random walk and a distribution of step lengths that followed either Brownian or Lévy distributions (Figure 2b,c, Supplementary Figure S1). Each simulation included five prey of each movement type, and prey were otherwise identical in appearance. When projected on the screen, the prey appeared as white dots moving at a constant speed (17 mms^−1^) and size (3 mm diameter), and could move within an area of 66 × 28 cm (width × height). Trajectories were long enough to generate 20-min videos at 60 frames per second. Each trial used a unique simulation of the prey to avoid pseudoreplication.

### Experimental procedure

All procedures were approved by the University of Bristol ethical review group (UIN/21/003). Fifteen fish were haphazardly netted from stock tanks the day before testing and moved to the holding area of the test tank (Figure 1). The next day, the simulation projection and camcorder recording for that trial were started, and a single fish was netted from the holding area to the test arena. Trials were ended when the fish made their first attack or 15 min had elapsed without an attack. The standard body length of the test fish was measured using callipers to the nearest millimeter after each trial. Each fish was tested only once. Trials were conducted between the 9th and 26th November 2021.

### Data extraction and statistical analysis

From the camcorder recordings, the frame at which the fish made their first attack was identified. Only the first attack was included in the analysis. The identity of the prey (Brownian or Lévy) was determined by visual comparison of which prey was attacked in the video to the plotted prey coordinates from the simulation at the corresponding time step. An exact binomial test was used to test whether the frequency of attacking each prey type differed from the random expectation of 0.5, as the two prey types were equally common. A binomial Generalized Linear Mixed Model (GLMM) using the lme4 package (Bates et al. 2015) was used to determine whether the fish’s standard body length or the trial order had significant main effects on the probability that either Brownian or Lévy prey were attacked using the drop1() function. The date of each trial was included as a random effect to control for multiple trials being carried out on the same day.

The exact binomial test and GLMM test whether Brownian or Lévy prey were preferentially targeted, and whether there are explanatory variables that affect this preference. In the second stage of the analysis, we conducted randomization tests to determine whether the movement paths of the prey could directly explain the preference for a particular prey type. The difference in Brownian versus Lévy motion is the frequency and duration with which individuals move in a straight line (Figure 2b,c, Supplementary Figure S2). The first randomization tested whether the prey that was targeted was turning less or more than that prey had done earlier in the trial. Specifically, we tested whether the turning angle of the targeted prey was greater or less than expected from the predators attacking that prey at randomly chosen times in that trial before the attack (i.e., whether the predators preferentially attacked that prey when they were moving in straighter or more sinuous paths than usual; see Supplementary Figures S2, S3, and S4 for details).

A second randomization procedure tested whether the predators had a preference between the prey based on their turning behavior just before the attack. This involved testing whether the turning angle of the attacked prey was greater or less than expected if the predators randomly chose a prey to attack at the same time step that the observed attack occurred (Supplementary Figures S2 and S5). All statistical analyses were carried out in R version 4.1.2 (R Core Team 2019). The simulations and statistical analysis can be recreated from the R code and experimental data provided as Supplementary Material.

## RESULTS

In 54 of the 117 trials, the test fish made an attack within the 15-min trial time. Data from two of these trials were lost due to technical malfunctions. In 35 of the remaining 52 trials with attacks (67%), the fish attacked a prey with Lévy motion (Figure 2d; exact binomial test: *P* = 0.018). The standard body length of the fish and the order of testing had no effect on whether Brownian or Lévy prey were attacked (binomial GLMM: body length: LRT = 0.0042, *P* = 0.95; test order: LRT = 0.018, *P* = 0.89).

There was no evidence from the randomization tests that the fish preferentially targeted the attacked prey when it was moving in a more or less straight path than expected from randomly timed attacks on that prey (Supplementary Tables S1 and S2, Supplementary Figure S4). In contrast, there was a statistically significant tendency for the attacked prey to have straighter movement in the time period immediately before the attack than expected if the predators selected a prey randomly at the same moment as the attack (Table 1, Supplementary Figure S5).

**Table 1:** The quantiles of the observed proportion of turns (mean value across the 52 trials) and observed mean angle turned (mean value across the 52 trials) in the corresponding distributions of expected values if the fish attacked a randomly selected prey at the same moment as the observed attacks

Time window (frames)	Quantile of mean observed proportion of turns in expected distribution	Quantile of mean observed mean turn angle in expected distribution
60	**0.014**	**0.018**
120	**0.024**	**0.023**
180	0.027	**0.013**
240	**0.019**	0.028
300	**0.012**	**0.018**

As two-tailed tests, the quantiles are statistically significant (marked in bold) if they are less than 0.025 (the attacked prey turned less than expected) or greater than 0.975 (they turned more than expected). See also Supplementary Figure S5.

## DISCUSSION

The fish we used as predators preferentially attacked those prey with Lévy motion twice as often as those with Brownian motion. Our randomization tests found no evidence that attacks on prey were more likely when they were engaged in straight movements (i.e., with low turning), suggesting that the timing of attacks was not influenced by prey behavior. However, when the fish did attack, they preferentially targeted those prey that were turning less than other prey; as prey with Lévy motion were more likely to be turning less, this can explain the preferential targeting of prey with Lévy motion. Exploring how general the effects reported here are across predators and contexts would be the next step. The advantage of a virtual prey system allowing for precise control over prey traits and hence minimizing confounding effects is, however, countered by the limited range of predators that are suitable for testing in such an artificial, laboratory-based set up. Another valuable extension of the results here would be to explore the consequence of allowing predators repeated exposure to prey that vary in their movement pattern, as recently we have shown that predators can adapt to unpredictability of fleeing prey (Szopa-Comley and Ioannou 2022). Any such future work would ideally not only consider the choice of which prey is targeted among those displaying different types of motion, but also allow the prey to be captured and consumed so that attack success can be measured.

Although we find there may be a cost to Lévy motion, Lévy motion might still be selected for if it is a sufficiently better search strategy to find resources (Viswanathan et al. 1999; Bartumeus et al. 2002; Humphries et al. 2012; Guinard and Korman 2021). For cases where there is a maximum rate of resource use (for example, limited by rates of digesting food [Papanikolaou et al. 2014]), a reduced time searching for resources using efficient, but potentially higher risk, Lévy search would allow for more time spent safe in refuges. Future work could explore the conditions that affect the trade-off between resource acquisition and predation risk, including how the total predation risk and how it changes over time affects movement trajectories. Our experiment was designed to test the predation risk faced by prey using Lévy motion in contrast to Brownian motion as comparisons between these two search patterns are made frequently in the animal movement literature (Bartumeus et al. 2002; Humphries et al. 2010; Sims et al. 2012; de Jager et al. 2013; Abe and Shimada 2015). However, other movement patterns are thought to be common, such as correlated random walks (Codling et al. 2008; Reynolds 2010; Auger-Méthé et al. 2015; Bailey et al. 2018), and these movement patterns may provide a more favorable trade-off between foraging success and predation risk than either Lévy or Brownian motion.

Another extension of this study would be to explore how movement behavior of prey interacts with other aspects of their anti-predatory defenses, such as living in groups (Ioannou 2021). Camouflage is a very common anti-predator trait (Cuthill 2019), and movement is known to adversely affect camouflage (Ioannou and Krause 2009). However, it remains unexplored how details of movement (e.g., speed, predictability, rate of change in direction) quantitively affect ease of detection by predators, and how consistent such trends would be across different types of camouflage. However, as well as camouflage, there is mounting evidence that the appearance of moving prey can influence the ability of predators to estimate the speed and direction of prey (Scott-Samuel et al. 2011; Stevens et al. 2011). It may be that patterning in prey might modify the targeting preferences explored here and/or any effects of movement pattern on the subsequent success of attacks by predators.

Previous consideration of Lévy motion has focused almost exclusively on the consequences for foraging. Here, we show that there may be consequences for predation risk driven by differential attack probabilities by predators. Since most foragers have to contend with potential predators, we hope that our work encourages a broader perspective in future study of Lévy motion patterns in particular, and search behavior in animals more generally.

## Supplementary Material

arad039_suppl_Supplementary_Material_S1

arad039_suppl_Supplementary_Material_S2

arad039_suppl_Supplementary_Material_S3

## Data Availability

Analyses reported in this article can be reproduced using the data and R script provided by Ioannou et al. (2023).
